# Report on Dental Education

**Published:** 1875-04

**Authors:** J. A. Watling


### REPORT ON DENTAL EDUCATION.

BY J. A. WATLING, D. D. S.

*Read before the Michigan State Dental Society.*

As Chairman of your Committee on Dental Education I submit for your consideration a few brief remarks.

This Association in 1866 adopted as a proviso, to Art. 8th Sec. 1, the following Resolution: "That no person shall be eligible to membership in this Association, who commences the practice of dentistry, after January 1st 1866, unless he be a grвduate of a legally authorized dental college." My object in the allusion, is to show the progress that the profession of dentistry has made since that time. Many of you will remember that at the time of its adoption, this resolution was strongly contested by some on the ground of its being impolitic, fearing that we old fellows would in time all die out, and not enough of the rising generation appear with the degree of D. D. S. to fill our places, and consequently the Michigan Dental Association sink into oblivion for want of membership But as an assocition, we are to-day flourishing, notwithstanding this resolution, and we think with regard to it, that it has done very much toward accomplishing the end sought, viz: that of stimulating young men to prepare themselves for the thorough practice of our profession in dental colleges.

The year following, this action was fortified by the Ohio Dental Association, adopting the same. Eight years have passed and now we see with pleasure, that the American Dental Association about to adopt an article similar for its constitution: that of admitting only graduates to membership in the future, and your Committee would here advise that the delegates from this Association to that body be instructed to vote and work for the adoption of such an amendment. It is time our profession cease to recognize any young man introducing himself to the pnblic as a dentist, unless he has earned a diploma from some of our numerous dental or medical colleges. And just here let me suggest a change in our constitution of Art. 8th, Sec. 1st, last line, by inserting the words "or medical," so that it shall read, "That no person shall be eligible to membership in the Association, who commences the practice of dentistry after January 1st, 1866, unless he be a graduate of a legally authorized dental or medical college." I do this believing a student who has received a thorough medical education, and has had a good dental preceptorship is as well qualified for practice as one who has received the regular dental degree, and I have entertained the question a long time, whether it would not have been better for us had we never cut loose from the mother profession, but remained with her as the oculist and aurist have done; only establishing such chairs as are necessary for the specialty and it is with great interest that we note a growing disposition in that direction. There can be no doubt but that a practitioner of dentistry should attain as thorough medical knowledge as one who confines his attention to the diseases of the eye and ear. I think the profession has suffered great injustice at the hands of the Regents of our University in their not establishing a chair of dentistry long ago. It is within the remembrance of some of you, when it was promised us, as soon as the nescessary funds should be at their disposal. No doubt it will require some increase of expense, most new departures do, but how long did that deter them from admitting ladies at an even greater expense, How few people realize the injury they do themselves by entrusting their teeth to ignorant practitioners, injuring, almost always, irremediable.

Thorough education is absolutely necessary to successful dentistry; it matters not how trival the operation. But with the open doors of the University there is not the slightest excuse for a young man to offer his services to the public, until he has graduated. We notice a strong feeling among the members of the American Dental Association, to increase the the term of studentship from two to three years, in this we would fully concur. Yet, how can a preceptor say to his student, that he will exact three years study of him when dental colleges require but two years at the most, and some of these have been known to graduate extra smart students in much less time. The colleges are the levelers of the standard of the profession. If they will declare for a studentship of three years, the profession will at once acquiesce in the decree, and in no case are they excusable for granting honorary degrees, except where eminence has been attained by long years of toil and study. We can cite cases where young men have bought their diplomas. In such cases money was the only real qualification, and yet at home, they parade these diplomas to the public in a way that would, make a regular graduate who has, by attainments and merit received his, blush with shame, these things are very discouraging to those who have spent time as well as money and toiled hard for their well earned degree. I am of the opinion that colleges should be more rigid in the enforcement of their rules, and that students should be required to attend the full course of lectures or, in other words they should not be admitted after the course has begun, nor allowed to absent themselves during the term; it would be better also to increase the course to fully five months, with at least four lectures per day. A spring and fall course is a mere farce. so long as it is optional with the students whether they attend them. One suggestion and I will close, there is perhaps no part of the student's education more neglected in dental schools than a proper training in professional bearing and etiquette. On this subject, they should receive thorough instruction, they should be taught that they are entering upon a professional life not a trade; and that which would be admissible in a trademan's way of doing business is in direct violation of our code of ethics. Let our dental colleges be more thorough in this one particular, and we shall hear less complaint of dental quackery and the profession will have gained one step more in the standard of professions.

